# Aging population and digital inclusive finance, a natural experiment from china

**DOI:** 10.1371/journal.pone.0287292

**Published:** 2023-11-27

**Authors:** Jinghong Zhou, Xixi Ye

**Affiliations:** 1 School of Finance, Taxation and Public Administration, Shanghai Lixin University of Accounting and Finance, Shanghai, China; 2 School of Digital Economy & Trade, Wenzhou Polytechnic, Wenzhou, China; 3 School of Finance, Shanghai University of Finance and Economics, Shanghai, China; Nanjing Audit University, CHINA

## Abstract

This paper studies the role played by the digitization level of inclusive finance for the aging population. We leverage an unexpected Chinese national strategy that promotes mobile internet and other internet related integration in China to identify the impact of changes in state policies on the digitization level for inclusive finance in a regression discontinuity design. Although aging population is negatively correlated with the digitization level of inclusive finance, our empirical analysis reveals that the policy shock significantly increased the digitization level of inclusive finance among the aging population. We also find two opposite mechanisms: the income effect and the caring effect. Finally, our study suggests that the economic and social outcomes of the digitization level among aging population are bifurcated: happiness decreased but real estate investment increased.

## 1. Introduction

According to the United Nations forecast, the proportion of the Chinese population aged 65 and above is expected to increase from 14 percent in 2022 to 30 percent in 2050 [1]. The substantial increase in the proportion of older adults within the population implies a rapidly expanding aging population in the upcoming decades. As China, along with many other regions around the world, grapples with challenges posed by an aging population and declining birth rates, digital solutions can play a critical role in ensuring social and economic well-being for older adults. In 2015, Premier Li Keqiang signed the first "Internet+" national strategic plan, which local governments subsequently implemented. This close relationship between the aging population and the level of digitization in inclusive finance through the "Internet+" has attracted attention from policymakers and researchers alike. The importance of studying this topic is evident given the significant impact of an aging population on society and the economy. As such, it is essential to explore the potential of digital solutions, such as the "Internet+", in addressing the challenges of aging populations. By doing so, we can create a more inclusive and sustainable society for older adults.

China is entering an era of aging population, but many seniors are still struggling to keep pace with the digital age. While traditional finance theory suggests that digital finance is the second-best option for users, it has become the primary platform for individual financings, such as Alipay. According to Lin Bin [2], Ali’s Chief Customer Officer for the elderly group customer service, the most common request from elderly people is for their children to be as patient as him. Alipay’s 2020 Senior Citizens’ Digital Life Report [3] highlights that the number of elderly users of Alipay has increased by 4.5 times from 2017 to 2020, with even higher growth rates observed in less-developed areas, reaching 5.5 times. The report further reveals that seniors prefer to use Alipay for investments and social networking, rather than payment. Among the various services of Alipay, the three most commonly used by the elderly are wealth management, social networking, and payment.

Given the increasing aging population globally, understanding the interaction between the aging population and digital finance is crucial. Digital inclusive finance refers to the use of digital technologies and financial services to provide access to banking and financial services for individuals and communities who are typically excluded from traditional financial systems. This includes people who live in rural or remote areas, those who are unbanked or underbanked, low-income households, and marginalized groups.

The digitization level of inclusive finance is a subset of digital inclusive finance. It refers to the degree to which financial services and transactions are conducted through digital channels, such as mobile banking, online banking, electronic payments, and other digital financial tools. A high level of digitization means that financial services are more accessible, efficient, and cost-effective, which can enhance financial inclusion and alleviate poverty. The digitization level of inclusive finance is of paramount importance since it has the potential to transform the lives of millions of individuals who are currently excluded from the formal financial system. Digital technologies can reduce the costs and obstacles associated with traditional banking, thereby making it easier for people to save, invest, and access credit. This can have a positive impact on economic growth, development, as well as individual well-being and quality of life.

In addition, the digitization of financial services can also increase transparency and accountability, reducing the risk of fraud and corruption. This can help to build trust in financial systems and institutions, which is essential for sustainable economic growth and development. Overall, the digital inclusive finance has the potential to improve financial inclusion, reduce poverty, and promote sustainable development.

The potential consequences of digital finance for the aging population are significant, but poorly understood. While there is a growing body of literature on digital inclusive finance and its impact on economic growth, income inequality, and traditional financial services, little is known about how the aging population interacts with digital finance. This research tries to fill the research gap by exploring the potential social and economic outcomes caused by digital inclusive finance among the aging population.

The motivation behind this paper is to explore the impact of digital inclusive finance on the aging population, given the surge in the elderly population and the increasing importance of digital solutions in ensuring their social and economic well-being. While several studies have examined the implications of digital finance inclusion on the Chinese economy [4–7], little is known about its effects on the elderly population. Existing literature presents divergent views on how the aging population interacts with inclusive finance. Some scholars suggest that the development of inclusive finance can positively impact the elderly population, but only after the population of the elderly exceeds a threshold level [8]. Others argue that inclusive finance negatively affects the elderly because digital finance is not user-friendly for them and requires financial resources [9, 10]. Given the lack of consensus in the existing literature, our paper aims to investigate the relationship between the provincial digitization level of inclusive finance and the aging population. Specifically, we examine whether the "Internet+" strategy can work as expected. To address this question, we study the impact of an exogenous policy shock on the relationship between the digitization level of inclusive finance and the aging population. Our study contributes to the literature by providing insights into the effectiveness of digital inclusive finance for the elderly population in China. By understanding the impact of digital inclusive finance on the aging population, policymakers and financial service providers can develop effective and user-friendly digital finance solutions tailored to the needs of this demographic.

The main findings of this study are as follows. First, the aging population negatively correlated with improved levels of digitization, but the implementation of the "Internet+" policy at the provincial level significantly increased the level of digitization among the aging population. Secondly, we have identified two mechanisms underlying this relationship—the income effect and the caring effect. Specifically, the income effect suggests that provinces with a higher number of elderly individuals see a larger increase in disposable income and a larger increase in digitization levels after the implementation of the "Internet+" policy. On the other hand, the caring effect suggests that the digitization level is reduced in provinces with a high elderly dependency rate as more support provided by younger generations substitute the need for digital finance products. Finally, our regression analysis reveals that, socially, a higher level of digitization is associated with a reduction in the level of happiness among the elderly, while economically, a higher level of digitization is associated with an increase in digitization payment flowing into the real estate market.

This paper makes three main contributions. Firstly, while most existing literature on digital finance inclusion mainly focuses on economic growth [11–13], our paper expands upon current literature by examining the relationship between the digitization level of inclusive finance and the elderly population. Few studies have investigated this relationship, making our paper a valuable addition to the existing body of literature. Secondly, this paper also enriches the dimensions of the factors affecting the digital finance of the elderly. Our analysis not only considers the income effect, but also considers the caring effect among the elderly. By examining how care provided by younger generations can substitute the need for digital finance products, we provide a more comprehensive understanding of the factors that affect the adoption of digital finance among the elderly. The third contribution of this paper is that it analyzes the economic and social consequences of the digitization level of inclusive finance. Our empirical findings provide insights into the efforts to improve the well-being of the elderly and enrich the current digital finance literacy. Additionally, our research sheds light on how digital inclusive finance can impact the real estate market economically and how it can reduce the elderly’s level of happiness socially.

This paper is organized as follows. Section 2 discusses the “Internet+” strategy and literature view. Section 4 explains the data and builds on the empirical model. This section also furnishes a related theories and hypothesis. Section 5 explores the empirical analysis and illustrates the results. The last section concludes our findings and provides policy suggestions.

## 2. The event and literature review

### 2.1 “Internet+” event: An overview

The “Internet+” strategy emphasizes the integration and innovation of a new generation of information technology, represented by cloud computing, the internet, and big data, with modern manufacturing and producer services. The strategy aims to create an environment for mass entrepreneurship and innovation, support industrial intelligence, enhance new economic development momentum, and promote the quality, efficiency, and upgrading of the national economy.

**Fig 1:**
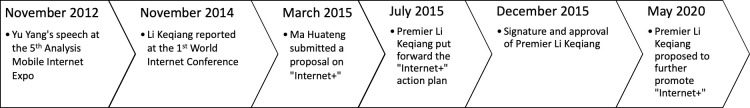
Event timeline.

On March 5, 2015, during the third session of the 12th National People’s Congress, Premier Li Keqiang introduced the “Internet+” strategy for the first time in the nation’s central government work report (Fig 1). The "Internet+" strategy has since become a crucial means of boosting the economy, promoting reform, and enhancing people’s well-being. The national strategy was then conveyed to the provincial governments and incorporated into the provincial projects.

The concept of "Internet+" in China originated from a speech delivered by Yu Yang at the 5th Analysis Mobile Internet Expo in 2012, where he introduced the idea of applying the "Internet+" to various industries by combining multi-screen, full-network, and cross-platform user scenarios. The government subsequently embraced this concept, with Premier Li Keqiang proposing the "Internet+" action plan in the government work report on March 5, 2015. This plan aimed to promote the integration of mobile internet, cloud computing, big data, and the internet advancement with modern manufacturing, and guide internet companies to expand their international market. Followingly, the State Council issued the "Guiding Opinions on Actively Promoting the ’Internet+" action to accelerate the improvement of the level of industrial development and enhance the innovation capabilities of various industries. The "Internet+" concept represents a practical achievement of internet thinking, which uses information and communication technology and internet platforms to deeply integrate the internet with traditional industries and create a new development ecology.

## 3. Literature review

### 3.1 Digital inclusive finance and aging population

#### 3.1.1 Inclusive finance

A significant number of literatures have investigated digital inclusive finance, including the measurement and analysis of the modern Chinese digital inclusive finance [8, 14]. Demirguc-Kunt et al. [15] aimed to measure financial inclusion, and they found that mobile money accounts have become increasingly prevalent in many countries and can provide a pathway to financial inclusion for the unbanked. Other studies have explored the impact of digital inclusive finance on economic growth and income inequality [4, 6, 16, 17], as well as its potential substitution effect on traditional financial services [18, 19]. Park and Mercado [20] found that financial inclusion has a significant and positive impact on poverty reduction, particularly in countries with lower levels of financial development. However, there is limited research on how the aging population interacts with digital finance.

#### 3.1.2 Aging population finance

The aging population is a growing concern in many countries as it is associated with increased public financing costs, political pressure and impedes economic growth [21–23]. Despite the importance of this issue, previous studies have not explored the relationship between digital finance and the aging population. A small group of literature studies have investigated the impact of digital finance on the aging population, but these studies have primarily focused on fraud prevention [24] and the exposure effects [25]. However, these studies were limited to Chinese literature and were only able to establish a correlation between aging population finance and digital inclusive finance, due to a lack of exogenous shocks.

### 3.2 Income, caring and digitization

Two theories are closely related to how digitization finance affects investors’ decisions. The first is the income effect theory, which was first introduced by Eugen Slutsky in 1915. According to this theory, when an individual’s income increases, their purchasing power increases, leading to a rise in demand for goods and services. The income effect implies that an increase in the aging population’s disposable income can result in a change in demand for digitization finance, with a shift from the initial indifference curve to the final indifference curve. Several scholars have explored the relationship between the income effect and digital inclusive finance. Allen [26] discussed the income effect and its relevance to consumer demand for goods and services. Moscati [27] emphasized that the income effect is an essential concept in microeconomics, as it helps to explain changes in consumer behavior in response to changes in income.

The second theory is the substitution effect theory, which states that the substitution effect can be negative for consumers by reallocating resources between choices [28, 29]. The aging population’s demand for digital inclusive finance can decrease if other choices become more important. That means that when more caring is provided for the elderlies, other finance products become relatively more expensive, leading people to substitute some of the more expensive digital finance services for the now cheaper caring.

Both income effect theory and the substitution effect theory play a crucial role in understanding how digital inclusive finance affects the investment decisions of aging investors. By exploring the relationship between the income effect and digital inclusive finance, researchers can gain a better understanding of the factors that influence aging investors’ demand for digital inclusive finance. Thus, further research is needed to understand the extent to which the income effect influences the demand for digital inclusive finance among aging investors.

### 3.3 Happiness, realty investment and digitization

Existing literature has produced conflicting results regarding the impact of digital finance on happiness. Some researchers argue that the relationship between digital finance and subjective happiness is U-shaped, depending on the stage of digital finance [30]. According to the U-shaped supporters, a low level of digital finance in the early stages reduces residents’ happiness, while a high level of digital finance in the middle and later stages can effectively improve happiness. Conversely, other researchers argue that the development of digital inclusive finance has significantly improved the happiness of younger age groups, but the impact on the happiness of the elderly presents an “inverted U-shaped” relationship that first rises and then declines [31]. Fang et al. found that a certain degree of education, high social interaction, and a preference for risk can moderate the reduction in elderly happiness, but, in general, the development of digital inclusive finance reduces the happiness of the elderly. Due to the conflicting results of existing literature, this paper aims to investigate if the aging population experiences greater happiness after the policy shock and if they benefit from the digital inclusive finance.

Existing studies have demonstrated that Chinese monetary policy, bullish stock market, and rising credit risk have a significant impact on the increase in real estate prices [7, 32, 33]. Our research contributes to this body of the literature by showing that digital payment adoption through inclusive finance can also lead to an increase in Chinese real estate consumption. The driving force behind this trend is the absence of alternative investment opportunities beyond real estate [34].

### 3.4 Research gap

Finally, the current literature lacks research on the impact of digital finance in the event study of “Internet+”. In fact, aging population finance is a crucial aspect of public welfare [2, 35], particularly as China’s long-term care system faces challenges from declining birth rates and an aging population [36]. This study aims to contribute to the existing scholarship by examining the causal relationship between the “Internet+” strategy and the digital financial market among the aging population.

To the best of our knowledge, few studies have explored policy shocks to the aging population in the digital financial market, and most of them are only theoretical. As such, the causal relationship between the aging population and the impact of digital inclusive finance remains unclear. Also, this study aims to address this gap by uncovering the hidden mechanism of policy shocks as well as its empirical application to the digital financial market. Unlike the previous literature on similar topics, this study provides detailed empirical analysis to illustrate the impact mechanism of a shock resulting from an “Internet+” to the aging population. In addition, our empirical section offers a comprehensive understanding and regression analysis on how the digitization level of inclusive finance among the aging population is influenced by “Internet+”.

## 4. Data and methodology

### 4.1 Data

To test the theoretical hypothesis proposed in the second part, we construct a panel dataset of 31 provinces in China from 2011 to 2020. The main source of data is China’s National Bureau of Statistics census data. We use the census data to construct our main independent variable, *Elderly*, which is the population of age 65 and above elderlies per 100 thousand.

Our second source of data comes from the Digital Inclusive Finance Index (DIF) developed by the Digital Research Center of Peking University in collaboration with Ant Financial. The index is a comprehensive indicator that measures the level of digital development in finance and provides insights into the extent and depth of digital financial inclusion in various regions of China. The DIF is constructed using Alipay transaction account data and covers 31 mainland provinces, 337 cities above the prefectural level, and around 2,800 counties. We focus on the provincial-level data from the DIF as a crucial benchmark for evaluating the digital financial landscape in China. Our data sources enable us to reflect the dynamic changes in digital inclusive finance development across different provinces. The DIF is composed of three main parameters—coverage breadth, usage depth, and digitization level of inclusive finance.

In this paper, we explore the *Digitization* level of inclusive finance as the independent variable. The *Digitization* level of inclusive finance parameter comprises four business classification indexes: mobility, convenience, cost, and credibility, which are crucial factors influencing users’ adoption and usage of digital financial services. The *Digitization* level of inclusive finance considers several factors, including the proportion of mobile payment transactions, the cost of financial services, and the level of credit that influence the adoption and usage of digital financial services. A higher *Digitization* level of inclusive finance indicates a more advanced levels of financial inclusion and a more frequently usage of digital payments. This parameter indicates how credible, accessible, affordable, and mobile the inclusive finance is in a given province and year. It measures the extent of digital financial services that are inclusive and accessible to a broad population, particularly those who are underserved or excluded from traditional financial services. This parameter is a significant component of the digital inclusive finance index system. Specifically, greater convenience in digital financial services leads to a higher proportion of mobile payment transactions relative to total payment transactions, which results in lower costs, such as lower interest rates on consumer loans and loans for small and micro-enterprises. And a higher level of credit means a greater proportion of deposit-free payment transactions relative to total payment transactions. Thus, the value of digital inclusive finance is therefore better reflected by this *Digitization* level of inclusive finance parameter.

The third data source for this study is the provincial government annual work report, which is an important government document in China. Each province provides a report on their work performance, achievements, problems, work guidelines, tasks, and measures for the next year at the annual government work report conference. The report is presented to the public, National People’s Congress representatives, and members of the Chinese People’s Political Consultative Conference. As a comprehensive accountability report of the government’s work to the people and representatives, it enables the public to understand the effectiveness of the government’s work and the next step in the work focus. Representatives can use the report to supervise and provide suggestions on government work, while it also serves as a vital reference for formulating provincial government work plans and policies. Subsequently, we employ text analysis to construct our policy shock variable, *Shock*. This independent variable is based on the frequency of the term "Internet +" mentioned in the annual work reports, beginning with the year of its first appearance. *Shock* variable takes a value of 0 before the year 2015. Happiness data come from the Chinese General Social Survey (CGSS) and all other variables are collected from the census data.

### 4.2 Methodology

We evaluate the “Internet+” policy impact on the digitization level of inclusive finance among the aging population by the following panel fixed-effects regression model:

$${Digitization}_{it}=\beta_{1}{Shock}_{it}+{\beta_{2}Elderly}_{it}+\beta_{3}{Shock}_{it}*{Elderly}_{it}+X_{it}+T_{t}+\lambda_{i}$$ (1)

where *Digitization*_it_ represents the digitization level of digital inclusive finance at province i in year t. *Shock*_it_ means the frequencies of the word “Internet+” appeared in provincial government reports at province i year t. *X*_it_ is a series of provincial-level control variables that change over time and affect the level of digitization, including the logarithm of provincial GDP and the logarithm of population. $T_{t}$ represents a time-fixed effect, which can control for unobservable common shocks at the provincial level that change over time, such as long-term trends in the level of digitization. The provincial fixed effect *λ_i_* can control for time-invariant and unobservable state characteristics. This paper adopts the provincial-level clustering robust standard error estimation model (1) to solve the problem of heteroscedasticity between different provinces and intra-provincial serial correlation. The main focus of this paper is the coefficient β3 of Shock*Elderly, which captures the effect of “Internet+” strategy on the level of digitization among the elderly population. Standard errors are clustered at the province level.

We consider two potential mechanisms of “Internet+” strategy that affect the digitization level of inclusive finance among the elderly group. The first type is the income effect, which improves the digitization level. To measure the income effect, we use the indicator of per capita disposable *Income* of all residents from the census data, which refers to the average disposable income of residents divided by the number of permanent residents. This indicator of disposable income refers to the total amount of annual per-capita available final consumption spending and savings of residents within a particular province. And, the second category is the caring effect, which decreases the digitization level. To measure the caring effect, we use the dependency ratio of the elderly population from census data, which refers to the percentage of the elderly population to the working-age population in the total provincial population. *Eldercare* is a percentage of how many elderly people are taken care of by every 100 working-age population at the provincial level.

To investigate the economic and social consequences of the Internet+ strategy, we consider the following two categories. The first consequence involves real estate investment. 1. *Commercial Housing Sold*. Commercial Housings are a type of housing properties, which holders can trade and use with property rights; its opposite type is affordable housing. We use the amount of commercial housing sold (in one million RMB) during the reporting period. The sales amount includes the total contract price of commodity houses, which includes the secondary housing market sales and the sales of new houses. 2. *Residency Housing Sold*. We use the amount of residential housing sold (in one million RMB) during the reporting period. The sales amount of residential housing consists of two parts: the secondary housing market sales and the sales of new houses. Residential housing types include affordable housing, single housing, and high-end apartments. 3. *Business-Purpose Housing Sold*. We use the amount of business-purpose housing sold (in one million RMB) during the reporting period. The sales of business-purpose housing include: the secondary housing market sales and the sales of new houses. The types of business-purpose housing include resorts, restaurants, shops, sales offices, grain stores, bookstores, stores, restaurants, vegetable stores, gas stations, and other miscellaneous business places.

The second consequence involves social happiness. 1. *Happiness*. The average provincial happiness level, we use the micro-level data from CGSS. We aggregate the surveyed individual happiness level into annual provincial level. Because the CGSS’s latest data only covers up to the year 2017, so we refer to Chua et al. [37] to fill up the missing data on *Happiness* in 2019 and beyond. Despite utilizing previous year’s data to impute missing values, it is not possible for us to observe the happiness levels for the year 2011 in Inner Mongolia province, and from 2011 to 2018 in Xin Jiang, Tibet, and Hainan province. Therefore, the final observation for *Happiness* is 284. 2. *Happy Share*. To capture weighted elderly happiness, we further multiply the previous “Happiness” index with the provincial share of the aging population. Table 1 reports the descriptive statistics of the main variables.

**Table 1:** Summary statistics.

Variable	Description	Obs	Mean	STD	Min	Max
Digitization	The digitization level of inclusive finance	310	290.1	117.3	7.580	462.2
Elderly	Population of age 65 or above, in 100 thousand	310	0.1074	0.2553	0.0013	1.8
Shock	The frequencies of the word “Internet+” in provincial work reports	310	1.152	1.819	0	11
Eldercare	Elderly dependency rate (Lao Nian Ren Kou Fu Yang Bi) from census data	310	14.13	3.565	6.700	23.80
Income	Log (disposable income / population)	310	10.94	1.260	7.056	13.58
Payment	Payment index (Zhi Fu) from DIF data.	310	184.8	91.83	0	379.5
Realtysales	Sales amount (Shang Pin Fang) in 100 million RMB	310	3557	3712	6.340	22573
Residentsales	Sales amount (Zhu Zhai Shang Pin Fang) in 100 million RMB	310	3014	3240	5.730	19830
Commercesales	Sales amount (Shang Ye Yong Fang) in 100 million RMB	310	315.9	264.8	0.6100	1304
Happiness	Happiness level	284	7.653	20.53	0.0189	144.5
Happyshare	Happiness * Elderly/ Population	284	0.8315	2.330	0.0014	17.05
Lpop	Log population	310	-0.9865	1.219	-3.662	2.822
Lgdp	Log GDP	310	9.695	1.000	6.416	11.62
Year		310	2015.5	2.877	2011	2020
Province		310	38.87	16.43	11	65

### 4.3 Theoretical hypothesis

The rapidly growing aging population presents a significant challenge to Chinese society and its economy. By 2025, China is projected to become a moderately aging society, with 20 percent of its population aged 60 years and above. By 2035, China is expected to become a severely aging society, with 30 percent of its population aging over 60 years old. In 2019, the Chinese aging population reached 137 million, and from 2010 to 2019, the growth rate of the aging population (over the age of 65) in China was nearly ten times the growth rate of the total population during this period [38]. The current changes in age structure pose significant challenges to the sustainable development of inclusive finance. Our paper covers three hypotheses that stem from cohort effects, income effects, and care effects.

#### 4.3.1 Provinces with more elderly, have lower level of digitization

Aging in digital finance implies the presence of cohort effects, where a higher aging for a cohort results in the fewer savings and external balances, but more bonds and equities [39]. Empirical research has found evidence supporting this conclusion. Poterba and Samwick [40] observed cohort effects in net worth but not for financial assets. Similarly, Goyal [41] found cohort effects in equity markets, where outflows of equities are related to aging. Jain and Raman’s research [42] further supports that digital finance adoption has cohort effects, where perceived risk and benefits influence the elderly in adopting digital finance.

**Hypothesis 1:** Population aging has a negative impact on the digitization level of inclusive finance, but the “Internet+” strategy alleviates the negative impact, and its positive impact overcome the negative impact.

#### 4.3.2 Income effects and care effects in an aging society

Eugen Slutsky’s theorem suggests income effects, where a higher the elderly real income leads to a higher the level of consumer demand. Empirical research has found that digital finance increases household income [43–45]. However, some scholars argue an inverse causal relationship between local income level and the level of digital finance. Guo et al [25] and Jiao [46] found that areas with lower income have a higher growth of digital inclusive finance. This is because lower-income areas intend to benefit more from digital inclusive finance by gaining more innovation and start-up business opportunities, as well as more increased consumer consumption.

Secondly, care effects imply the presence of substitute effects. Recent research on digital finance among aging population found that a higher caring ratio, also known as the dependency ratio, results in a lower level of digital finance [25, 47]. As caring provided by younger generations substitutes the need for the elderly to use digital finance, the elderly can rely on the younger generation when needed.

**Hypothesis 2:** In higher-income provinces, the aging population has a positive impact on the digitization level of inclusive finance during the "Internet+" strategy. Conversely, in more caring provinces, the digitization level of inclusive finance during the "Internet+" strategy is not positively impacted by the aging population.

#### 4.3.3 Happiness and asset holdings in aging society

Currently, there are different opinions on how digital finance affects happiness. The first series of literature finds that digital finance, such as mobile payments and social media, increases individual happiness. Bekalu et al. [48] and Zhao et al.[49] suggest that mobile payments benefit the quality of life, reduce transaction costs, stimulate entrepreneurship, and increase social interactions. However, the second series of scholars find that digital finance increases the income gap, and more elderly are exposed to high risks of fraud. Meng and Xiao [50] conclude that digital finance decreases the level of happiness because digital finance increases debt burden and overspending. Our study supports the second works of literature.

The aging population in China is experiencing increased levels of unhappiness, which can be attributed to several factors. One factor is the growing digitization of financial services in urban areas, which may lead to confusion or frustration for elderly individuals who are not familiar with or comfortable using digital tools. In addition, living in urban areas can be expensive and exacerbate financial stress for elderly individuals living on fixed incomes, such as pensions or retirement savings. Access to affordable housing, healthcare, and other essential services can also be limited, further impacting the well-being and happiness levels of the elderly population.

Moreover, the lack of a robust social welfare system in China [51, 52] can leave many older adults struggling to meet their basic needs, including healthcare and housing. The one-child policy in China has also contributed to the challenges faced by the aging population, with many elderly individuals facing the prospect of aging alone, without the support of a large family network. This, combined with the increasing digitization of financial services, can lead to financial insecurity for the elderly, who may not have access to the same levels of family support or government assistance as they age. This can result in feelings of stress, anxiety, and uncertainty, which can impact their overall well-being and happiness levels.

Furthermore, elderly individuals in China may face difficulties adapting to new digital financial tools, such as mobile payment platforms, due to a lack of familiarity with technology or limited access to digital devices. This can lead to difficulties managing their finances, which can contribute to feelings of stress and insecurity. Additionally, the increasing digitization of financial services can contribute to feelings of loneliness and social isolation in the elderly population, which can further impact their happiness levels. Thus, the aging population in China is facing several challenges related to digital finance and the one-child policy, which are impacting their well-being and happiness levels.

As for asset holding, 72% of Chinese household wealth is in real estate properties [53]. Real estate investment in China is regarded with more stable returns than the stock market. As the population ages, the growing amount of population tends to be risk-averse and more likely to pay for real estate. Li et al.’s [54] empirical work found that real estate is a better investment option than other financial assets, as the aging population prefers lower risks. Li and his coauthors also show that the more the aging population a family has, the fewer risk assets the family hold and more real estate investment instead. Additionally, Saull et al. [55] found other supporting evidence that digital technologies make transactions faster and cheaper. For example, the Chinese digital finance platform Alipay provides links with auction houses, which significantly reduces the transactions and time costs with easier access.

One of the main reasons why older adults in China are buying more real estate is because of the lack of a well-developed social welfare system, such as comprehensive health insurance, pension plans, or social security, which has led many older adults to view real estate as a form of retirement savings. In China, older adults often choose to rent out secondary homes or bedrooms in their primary homes as a means of funding their retirement, especially given the limited investment options available to them. Additionally, in China, the one-child policy, which was in place until 2015, has resulted in many empty nesters, which refers to older adults whose children have moved out of their family home. These empty nesters may want to downsize or relocate to a more desirable location, which can drive demand for real estate. It is also worth noting that in China, real estate is seen as a symbol of wealth and status, particularly among the older generation. This can also drive demand for real estate among older adults. Overall, while the digital inclusive finance can make it easier for older adults to finance their real estate purchases, it may not be the primary driver of their increased purchases of real estate, particularly in China. The lack of a well-developed social welfare system and cultural factors can be the main drivers of this trend.

The rise of digital finance platforms, such as Alipay and WeChat Pay, has transformed the way elderly people in China purchase real estate. For instance, Maria Cordero [56], a grandmother of 15 grandchildren, chose to use Alipay to purchase a property on the mainland of China as affordable housing options were limited in Hong Kong. In July 2018, upon her arrival to the mainland, she instantly transferred a deposit of 80,000 yuan through Alipay to secure her desired property. This case study exemplifies how inclusive finance and the widespread use of smartphones among the elderly have facilitated property purchases for seniors.

According to the JD.com’s consumption data of the Double Ninth Festival in October 2019 [57], smartphones, smart canes, and other electronic products were among the top search keywords, underscoring the prevalence of technology among the elderly. Furthermore, this survey indicated that 44% of the new elderly individuals aged 55–65 who owned smartphones purchased them independently without any consultation from their family or friends. This indicates a growing trend of seniors embracing technology for financial transactions and purchases. With the increasing prevalence of smartphones among the elderly population, inclusive finance has expanded to include home-selling activities. One example is the Yu’ebao Home Purchase project, which was launched in March 2015 by Taobao Real Estate Channel and Fang.com [47]. This initiative offered 1,132 sets of properties in China’s top ten cities, including Beijing and Shanghai, to support Yu’ebao home purchases. Yu’ebao, a financial product of Alipay, was launched by Ant Group Co., Ltd. in June 2013 as a value-added service and demand fund management service product. With the Yu’ebao Home Purchase project, homebuyers can pay the down payment through Taobao, which will be frozen in Yu’ebao. Within three months after the down payment, the yield generated by the down payment in Yu’ebao belongs to the homebuyer, making it a convenient and profitable option for elderly individuals. The proliferation of digital finance platforms and inclusive finance initiatives has created a more accessible and streamlined property purchasing process for the elderly in China.

**Hypothesis 3:** The elderly population in a province with a higher level of digitization in inclusive finance tends to purchase more real estate during the "Internet+" strategy. However, this increased real estate acquisition does not translate to a higher level of happiness among the elderly population.

## 5. Empirical results

### 5.1 Baseline result

Table 2 reports our baseline regression results of the impact of “Internet+” strategy on the digitization level of inclusive finance among the aging population. Our result shows that the estimated coefficient of *Elderly* is negatively statistically significant at least at the 5% level. Column (3) shows the coefficient of the interaction term, Elderly* Shock is positive and statistically significant at least at the 5% level. Although more aging population means less digital inclusive finance, our study’s baseline results indicate that the "Internet+" initiative has a significant positive effect on the digitization level of inclusive finance among older adults.

**Table 2:** Baseline estimates of the impact of “internet+” on the digitization level of inclusive finance among aging population, by province, 2011–2020.

	(1)	(2)	(3)
	Digitization		
Elderly	-29.28***	-29.22***	-22.73**
	(8.743)	(8.699)	(8.948)
Shock		0.2404	-3.846***
		(1.585)	(1.233)
Elderly* Shock			89.76**
			(36.45)
Control Variables	Yes	Yes	Yes
Year Fixed Effect	Yes	Yes	Yes
Province Fixed Effect	Yes	Yes	Yes
*Adj R* ^2^	0.9749	0.9748	0.9759
*Within R* ^2^	0.0926	0.0929	0.1376
*N*	310	310	310

Standard errors in parentheses

* *p* < 0.1

** *p* < .05

*** *p* < .01

Specifically, column (1) shows a negative and statistically significant coefficient for *Elderly*, indicating that a higher proportion of elderly population is associated with lower levels of digitization in inclusive finance, after controlling for other factors. This suggests that the aging population may face barriers or challenges in adopting digital financial services. Column (3) includes an interaction term between *Elderly* and *Shock*. The coefficient for the interaction term is positive and statistically significant, indicating that the impact of the shock on digitization is stronger in provinces with a higher proportion of elderly population. This suggests that the policy may have been effective in promoting digital inclusive finance among the aging population.

On average, “Internet +” increases elderly digitization by 3.949 (89.76/22.73) times, compared to the sample mean if at least one province work report mentioned the keyword in the previous year, holding all other variables in the model constant. Our regression results support the hypothesis in section 4.

### 5.2 Regression discontinuity

**Fig 2:**
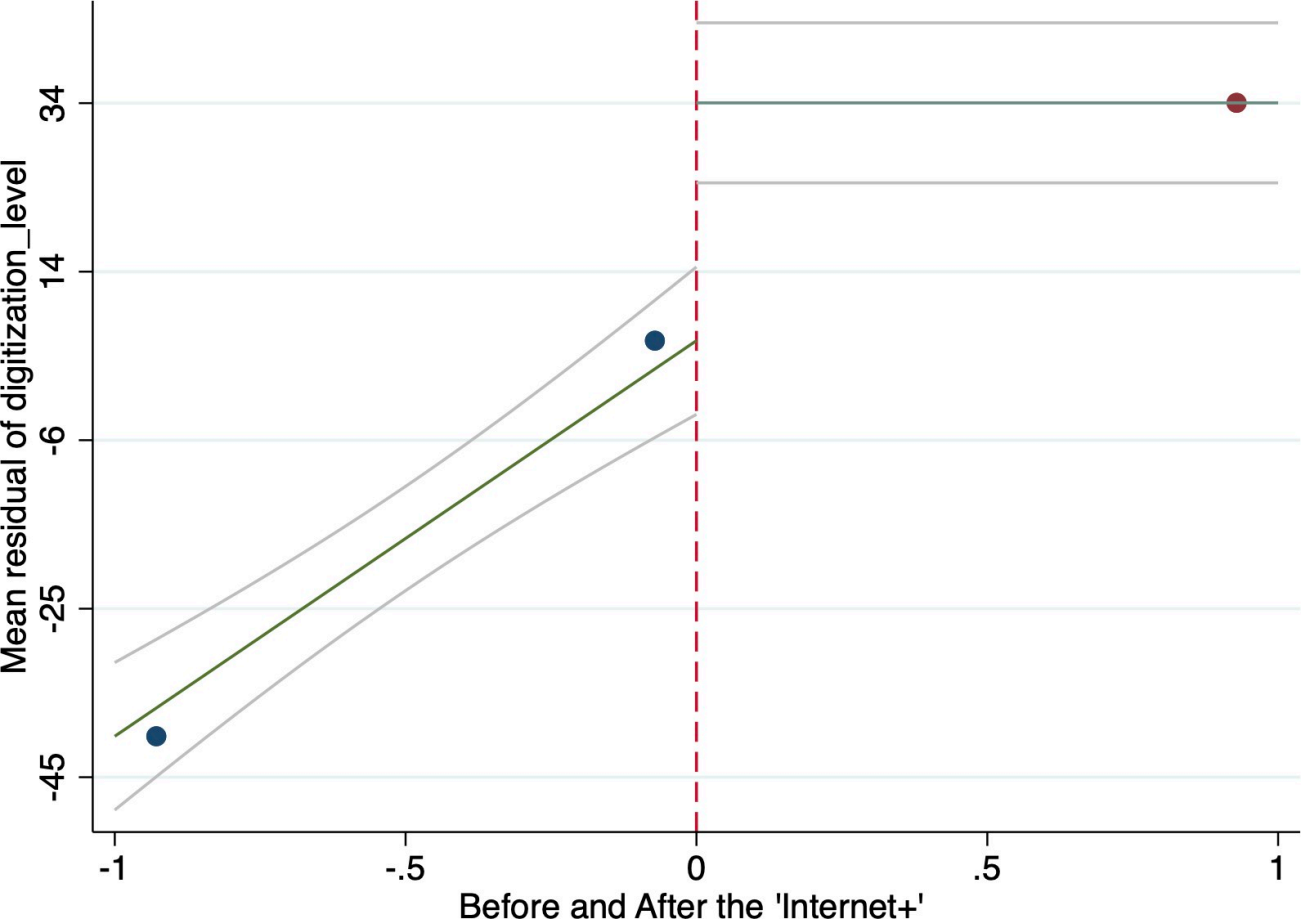
RDD.

In Table 3, we use the regression discontinuity (RDD) method to ease potential endogenous problem (Fig 2). Since the aging population who are the digital inclusive finance platform users cannot manipulate when the “Internet+” occurs, the running variables meet the premise of Lee and Lemieux [58] and Cattaneo et al. [59]. We estimate the optimal bandwidth for the regression function by using a local polynomial regression model. This involves estimating the regression function separately for different bandwidths around the cutoff point, and then choosing the bandwidth that gives the most stable and reliable estimate of the treatment effect. Table 3 shows the estimation results of the RDD estimation under the optimal bandwidth. The results show that the estimated coefficient of the policy shock dummy is significantly positive at the 1% statistical level, and the estimated coefficient value is 271.5, suggesting the “Internet+” strategy significantly increases the digitization level of inclusive finance.

**Table 3:** RDD estimation.

	(1)	(2)	(3)
	Digitization		
Shock	271.5***	271.5***	82.11***
	(4.400)	(4.400)	(28.47)
Control Variables	Yes	Yes	Yes
Year Fixed Effect	Yes	Yes	Yes
Province Fixed Effect	Yes	Yes	Yes
Bandwidth	1	1	7
Polynomial	1	1	2
kernel	triangular	epanechnikov	triangular
*N*	310	310	310

Standard errors in parentheses

* *p* < 0.1

** *p* < .05

*** *p* < .01

**Fig 3:**
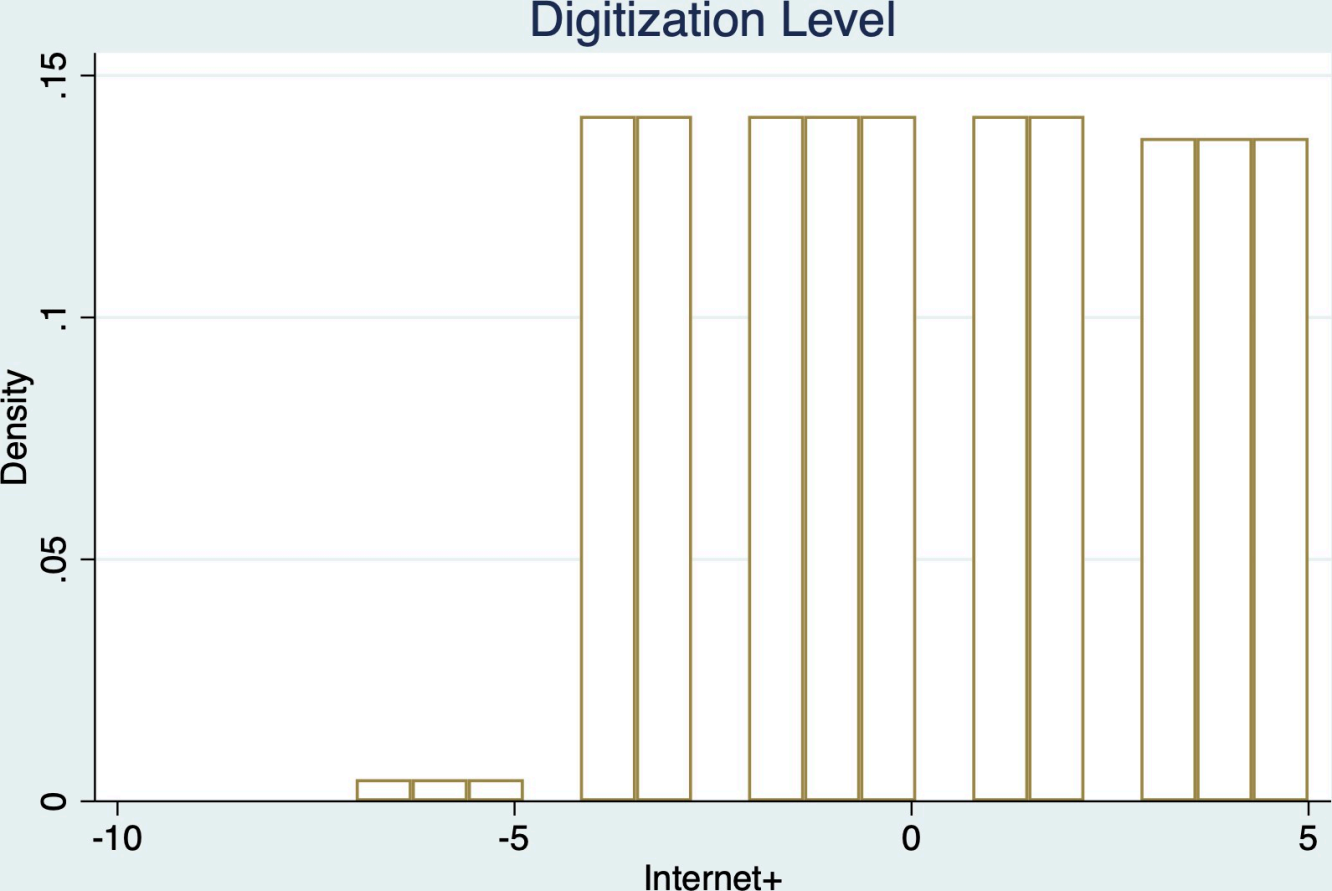
Density test.

The underlying logic of the density discontinuity test in Fig 3 is: if the micro-state cannot precisely control the value of its own driving variable, then the side of the breakpoint its driving variable falls on has a certain local randomness. This local randomness should ensure that it can be observed the continuity of the covariates around the cutoff point. Specifically, we grouped samples with a year between -1 (that is, the year before “Internet+” strategy is included in the provincial annual reports) and 1 (that is the year after “Internet+” strategy is included in the provincial annual reports) accordingly. From the Fig 3, we can see that provinces on both sides of the cutoff point are similar and do not show significant differences. The above evidence further strengthens our confidence in the effectiveness of our identification strategy.

### 5.3 Robustness

#### 5.3.1 Event study

**Fig 4:**
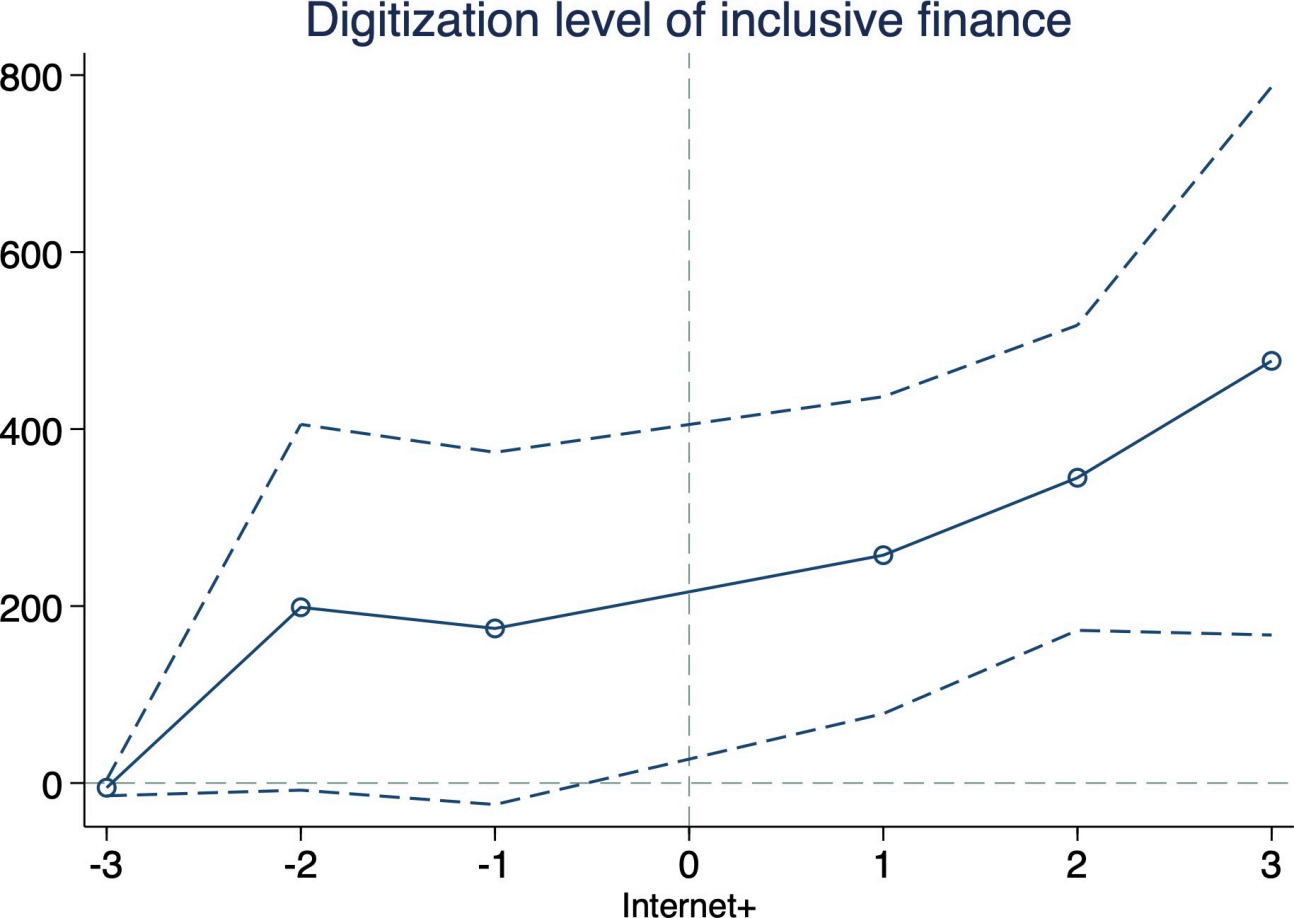
Event study. 95% confidence interval shown in dashes.

We use event studies to test the policy dynamic effects of the “Internet+”, and the results are shown in Fig 4. The results show that there was little significant difference in the digitization level of inclusive finance before the policy shock. Immediately after the “Internet+,” the policy effects were evident, demonstrating the robustness and reliability of the underlying model results.

#### 5.3.2 Placebo test

We further conduct a placebo test. Specifically, we change the occurrence of the “Internet+” event by 2–3 years. If the estimated coefficients of these pre-explained variables are not significant, it means that the benchmark regression results are effective. Table 4 shows the results of the placebo test, and none of the estimated coefficients of the pre-explained variables were significant.

**Table 4:** Placebo test.

	(1)	(2)
	Digitization	
DD_2012	130.9	
	(201.7)	
DD_2013		257.3
		(158.1)
Control Variables	Yes	Yes
Year Fixed Effect	Yes	Yes
Province Fixed Effect	Yes	Yes
*Adj R* ^2^	0.9749	0.9752
*Within R* ^2^	0.0951	0.1086
*N*	310	310

Standard errors in parentheses

* *p* < 0.1

** *p* < .05

*** *p* < .01

### 5.4 Mechanism

To test our first mechanism, we use the interaction term of disposable income, policy shock and the aging population. Table 5 reports the estimated income effect. The regression result shows a strong negative correlation between disposable income and the level of digitization of inclusive finance, suggesting that the aging population with higher incomes are more likely to use traditional banking methods, while those with lower incomes are more likely to rely on transactions and financial services through digital channels. And the interaction term of "Elderly*Shock*Income" is positively statistically significant at the 1% level, suggesting that higher income can accelerate the policy shock on the elderly population’s digitization level in inclusive finance. We further use the digital payment index from our DIF data to test the robustness of our income effect. Again, the interaction term of disposable income and the aging population is positively statistically significant at the 1% level. Thus, our empirical result indicates that elderly people with higher income are more likely to use digital payment methods even when facing a shock to digital inclusive finance.

**Table 5:** Mechanism: Income effect.

	(1)	(2)	(3)	(4)
	Digitization		Payment	
Income	-309.2***	-230.9**	-127.2*	-103.6
	(94.37)	(96.99)	(63.32)	(68.35)
Elderly		-24.75***		-2.109
		(8.971)		(3.176)
Shock		-6.137***		-1.749
		(1.835)		(1.140)
Elderly* Shock		-1281.1***		-190.4*
		(337.1)		(108.9)
Elderly* Shock* Income		130.1***		22.51**
		(31.94)		(10.48)
Control Variables	Yes	Yes	Yes	Yes
Year Fixed Effect	Yes	Yes	Yes	Yes
Province Fixed Effect	Yes	Yes	Yes	Yes
*Adj R* ^2^	0.9757	0.9793	0.9861	0.9867
*Within R* ^2^	0.1213	0.2646	0.0722	0.1239
*N*	310	310	310	310

Standard errors in parentheses

* *p* < 0.1

** *p* < .05

*** *p* < .01

Second, Table 6 reports the estimated caring effect. The "*Eldercare*" term represents the main effect of eldercare on the digitization level of inclusive finance, and the interaction term "Elderly*Shock*Eldercare" indicates the moderating effect of eldercare on the relationship between elderly population, shock, and digital inclusive finance. Overall, these results suggest that eldercare is an important factor for improving the digitization level of inclusive finance, especially for the elderly population in the context of a shock. The main effect of "Eldercare" on the digitization level of inclusive finance is statistically significant at least at the 5% level. And the interaction term is not positive, but is negative and statistically significant at the 5% level in column (4), indicating that the substitution impact on digital inclusive finance for the elderly is less severe in areas where there is more eldercare provided. Thus, our empirical result indicates that increased caring for the elderly is associated with a decrease in the use of digital payment among the elderly.

**Table 6:** Mechanism: Caring effect.

	(1)	(2)	(3)	(4)
	Digitization		Payment	
Eldercare	5.400**	3.851	2.712***	2.799***
	(2.156)	(2.551)	(0.8931)	(0.8960)
Elderly		-20.53**		-0.5855
		(8.976)		(3.095)
Shock		-2.834**		-1.740*
		(1.275)		(0.8604)
Elderly* Shock		-29.62		128.1***
		(74.76)		(29.13)
Elderly* Shock* Eldercare		5.667		-4.866**
		(4.506)		(1.875)
Control Variables	Yes	Yes	Yes	Yes
Year Fixed Effect	Yes	Yes	Yes	Yes
Province Fixed Effect	Yes	Yes	Yes	Yes
*Adj R* ^2^	0.9759	0.9775	0.9865	0.9871
*Within R* ^2^	0.1284	0.2008	0.0967	0.1502
*N*	310	310	310	310

Standard errors in parentheses

* *p* < 0.1

** *p* < .05

*** *p* < .01

### 5.5 Economic and social outcomes

Based on our previous analysis, the "Internet+" strategy has led to an increase in the digitization level of inclusive finance among the aging population, meaning elderly users increasingly adopting inclusive finance platforms for payment purposes. Given the importance of understanding the implications of this trend, our study explores the economic outcomes of this strategy on the aging population’s digital inclusive finance. We examine the economic outcome of real estate investment in Table 7, specifically looking at the sales of different types of properties in relation to the shock, elderly population, and digital payment. As per the regression results presented in Table 7, we find that the "Internet+" strategy has contributed to a rise in real estate property payments, and this can be attributed to the digital inclusive finance among the aging population. Our findings emphasize the importance of understanding the role of digital inclusive finance in affecting investment choices for the elderly.

**Table 7:** Economic outcome: Real estate investment.

	(1)	(2)	(3)	(4)	(5)	(6)
	RealtySales		ResidentSales		CommerceSales	
Elderly	-148.4	-208.2	-118.0	-173.5	-13.99	-14.59
	(294.1)	(236.2)	(284.5)	(226.4)	(19.87)	(20.07)
Shock	-609.4***	-389.3***	-550.8***	-351.9***	-28.96***	-23.77**
	(152.8)	(126.5)	(135.9)	(112.6)	(8.51)	(8.74)
Elderly* Shock	17165.7***	-25173.9***	15357.1***	-23107.0***	832.9***	-53.97
	(3577.3)	(6383.1)	(3230.0)	(5957.8)	(157.4)	(390.1)
Payment		38.20**		33.84**		1.232
		(16.47)		(14.04)		(0.977)
Elderly* Shock* Payment		136.9***		124.5***		2.794**
		(21.15)		(19.84)		(1.170)
Control Variables	Yes	Yes	Yes	Yes	Yes	Yes
Year Fixed Effect	Yes	Yes	Yes	Yes	Yes	Yes
Province Fixed Effect	Yes	Yes	Yes	Yes	Yes	Yes
*Adj R* ^2^	0.8654	0.9071	0.8590	0.9033	0.8785	0.8831
*Within R* ^2^	0.3177	0.5325	0.3099	0.5304	0.3198	0.3503
*N*	310	310	310	310	310	310

Standard errors in parentheses

* *p* < 0.1

** *p* < .05

*** *p* < .01

To explore the social impact of digital inclusive finance, we analyze the average happiness levels in each province and the interaction effect of these levels with the proportion of the aging population. The regression results in Table 8 indicate that the "Internet+" strategy is associated with a decrease in happiness levels. The interaction term “Elderly*Shock*Digitization” is negative and significant. This indicates that the negative effect of the "Internet+" strategy on happiness is stronger among the elderly population who use digital services. This information could be valuable for policymakers in designing and implementing policies that aim to increase the digitization of services while ensuring the well-being of the elderly population. Specifically, our findings suggest that digital inclusive finance among the elderly population is the underlying cause of this effect.

**Table 8:** Social outcome: Happiness.

	(1)	(2)	(3)	(4)
	Happiness		HappyShare	
Elderly	78.01***	78.04***	9.265***	9.324***
	(2.251)	(2.318)	(0.6945)	(0.6942)
Shock	0.3955***	0.3865***	0.0386**	0.0434*
	(0.1129)	(0.1244)	(0.0173)	(0.0223)
Elderly* Shock	-8.095***	7.181	-0.5883**	4.391**
	(1.680)	(6.994)	(0.2774)	(2.128)
Digitization		0.0012		0.0026
		(0.0079)		(0.0025)
Elderly* Shock*Digitization		-0.0413**		-0.0139**
		(0.0201)		(0.0062)
Control Variables	Yes	Yes	Yes	Yes
Year Fixed Effect	Yes	Yes	Yes	Yes
Province Fixed Effect	Yes	Yes	Yes	Yes
*Adj R* ^2^	0.9922	0.9922	0.9614	0.9618
*Within R* ^2^	0.9737	0.9739	0.8903	0.8923
*N*	284	284	284	284

Standard errors in parentheses

* *p* < 0.1

** *p* < .05

*** *p* < .01

## 6. Conclusion and implications

This paper presents a new perspective on the impact of the "Internet+" strategy on digital inclusive finance and the elderly population. Our analysis reveals a significant increase in the digitization level of inclusive finance among the elderly in China following the implementation of the "Internet+" strategy. Our regression discontinuity analysis confirms the robustness of this finding. We also identify two mechanisms through which the strategy affects digitization: income and caring effects. Notably, the increased digital finance inclusion during the "Internet+" era led to a substantial influx of capital into the real estate industry and an unexpected decrease in the happiness level of the elderly population. These findings contribute to the literature on digital finance inclusion and highlight the need for policymakers to carefully consider the broader social consequences of such initiatives.

The results of this study have significant policy implications. While the "Internet+" strategy has had a positive impact on various aspects of society and the economy, it has also led to an increase in hot money inflows into the real estate industry, with a negative impact on the happiness levels of the elderly population. Hence, it is crucial to understand the behavior and decision-making of the elderly under the influence of national policies. To mitigate the negative effects of this policy on the elderly, policymakers in China should focus on providing more public support services for low-income elderly groups and developing stable investment channels for middle-income elderly groups. Moreover, policymakers should implement regulations to prevent speculative behaviors associated with hot money inflows into the real estate industry. Furthermore, exploring the potential for developing new financial products and services that cater to the specific needs of the elderly population could substitute real estate investment for the aging population. Overall, these policy measures could help ensure that the benefits of national strategy are distributed more equitably among all members of society, including the elderly population.

One of the limitations of our study is that the estimated results from using the RDD method can only be interpreted as the causal effect of the treatment in a narrow range around the cutoff point. Therefore, caution should be taken when generalizing our findings to other parts of the distribution of the variable. Future research could explore the potential for stable investment options that substitute real estate investment for the aging population. One avenue worth exploring is the use of digital finance to provide alternative investment options that are less risky and more accessible to older adults. Furthermore, given the rapidly changing dynamics in the digital finance field, additional research on the impact of elderly happiness measures is necessary to provide more robust academic insights. Future studies could examine the relationship between digital finance inclusion and elderly happiness in different contexts and cultures, as well as the potential for digital finance to improve the well-being of older adults.
